# Activation of plantar flexor muscles is constrained by multiple muscle synergies rather than joint torques

**DOI:** 10.1371/journal.pone.0187587

**Published:** 2017-11-06

**Authors:** Takahito Suzuki, Ryuta Kinugasa, Senshi Fukashiro

**Affiliations:** 1 Department of Human Sciences, Kanagawa University, Yokohama, Kanagawa, Japan; 2 Department of Life Sciences, Graduate School of Arts and Sciences, The University of Tokyo, Tokyo, Japan; Duke University, UNITED STATES

## Abstract

Behavioral evidence has suggested that a small number of muscle synergies may be responsible for activating a variety of muscles. Nevertheless, such dimensionality reduction may also be explained using the perspective of alternative hypotheses, such as predictions based on linear combinations of joint torques multiplied by corresponding coefficients. To compare the explanatory capacity of these hypotheses for describing muscle activation, we enrolled 12 male volunteers who performed isometric plantar flexor contractions at 10–100% of maximum effort. During each plantar flexor contraction, the knee extensor muscles were isometrically contracted at 0%, 50%, or 100% of maximum effort. Electromyographic activity was recorded from the vastus lateralis, medial gastrocnemius (MG), lateral gastrocnemius (LG), and soleus muscles and quantified using the average rectified value (ARV). At lower plantar flexion torque, regression analysis identified a clear linear relationship between the MG and soleus ARVs and between the MG and LG ARVs, suggesting the presence of muscle synergy (*r*^2^ > 0.65). The contraction of the knee extensor muscles induced a significant change in the slope of this relationship for both pairs of muscles (MG × soleus, *P* = 0.002; MG × LG, *P* = 0.006). Similarly, the slope of the linear relationship between the plantar flexion torque and the ARV of the MG or soleus changed significantly with knee extensor contraction (*P* = 0.031 and *P* = 0.041, respectively). These results suggest that muscle synergies characterized by non-mechanical constraints are selectively recruited according to whether contraction of the knee extensor muscles is performed simultaneously, which is relatively consistent with the muscle synergy hypothesis.

## Introduction

The human musculoskeletal system comprises approximately 400 skeletal muscles [1] that provide flexible solutions to various complex movements. Consequently, a large number of degrees of freedom must be controlled by the central nervous system [2]. It has been proposed that the central nervous system addresses this problem by combining small groups of muscles [3,4] that are coactivated in a fixed intensity ratio [5,6]. Such groups of muscles are referred to as “muscle synergies” or “synchronous synergies” [7], although other definitions of synergy have also been proposed [8,9,10,11]. For example, a linear relation potentially suggestive of muscle synergy [4] exists between the activities of the brachialis and brachioradialis muscles, as the brachialis is consistently activated in proportion with the brachioradialis in all examined elbow actions [12]. Regression analysis has previously provided evidence for muscle synergies in simple cases. However, such analysis is unable to extract linear relationships from complicated activation patterns in which a single muscle can participate in multiple muscle synergies. To model these more complex situations, computational decomposition techniques such as non-negative matrix factorization have recently been applied for the identification of multiple linear relationships in muscle activities across different tasks [13,14,15,16]. These techniques have demonstrated that various movements can be described by a linear combination of a relatively small number of muscle synergies.

Despite numerous reports based on behavioral studies, the muscle synergy hypothesis in humans remains difficult to confirm or rebuke [7,17]. Even if muscles appear to be coactivated in a fixed ratio, as would be expected in muscle synergy, hypotheses unrelated to muscle synergies (e.g., minimization of energy) could also explain coactivation [12,18]. For example, previous studies have reported that the coactivation of muscles during walking could be categorized as muscle synergy [13,16,19,20]. However, synergy-like coactivation of muscles is also roughly predicted by walking simulations based on minimizing objective functions such as metabolic energy [21], the sum of squared muscle activations [22], or the sum of cubed muscle forces normalized by the physiological cross-sectional area [23]. Because these hypotheses distribute relative loads evenly to synergists, such models tend to predict that synergists are independently coactivated in a nearly fixed ratio during a specific movement, leading to misinterpretation as muscle synergy. Moreover, non-negative matrix factorization has previously been applied to extract muscle synergies from muscle activity patterns during an isometric force exertion task with a given posture where hip and knee sagittal plane torques (i.e., extension or flexion) were generated in various combinations [24]. However, in such a task, the activity of each muscle is approximately described by a linear combination of hip and knee joint torques where each joint torque has one coefficient for each muscle [25,26], without consideration to muscle synergies. Indeed, a previous study suggested that the matrix where muscle activities recorded in such tasks are combined for computational decomposition could itself be decomposed into a coefficient matrix and a torque matrix [18], with no relation to muscle synergies, although a similar coefficient matrix extracted by computational decomposition was elsewhere interpreted to represent muscle synergies [24]. Consequently, further evidence is required to verify the muscle synergy hypothesis in humans, and this evidence must be obtained in an experiment that compares the explanatory capacity of the muscle synergy hypothesis with that of other hypotheses.

A recent study suggested that muscle activity was not necessarily consistent with predictions made according to the above-mentioned non-synergy hypotheses. The minimization hypothesis predicts that the activity of the medial gastrocnemius muscle (MG), which also functions as a knee flexor muscle, and that of the soleus muscle (Sol) would decrease and increase, respectively, when isometric knee extensor contraction is added to isometric plantar flexor contraction. This prediction is only partly true, as it has been shown that MG activity decreases up to a point and then plateaus, while Sol activity consistently increases with increasing knee extensor muscle activity from medium to maximum levels [27]. Additionally, these findings indicated that MG activity does not always depend on the knee sagittal plane torque multiplied by a coefficient, in contrast with its behavior during tasks that combine hip and knee sagittal plane torques [25,26]. If the muscle synergy hypothesis can provide a better understanding of plantar flexor coactivation during plantar flexor contraction with or without knee extensor contraction, the muscle synergy hypothesis will be corroborated in terms of the linear relationships among muscle activities, as well as in terms of linear relationships between joint torques and muscle activities.

We hypothesized that the activity of each head of the triceps surae, i.e., the MG, lateral gastrocnemius muscle (LG), and Sol, would be determined by muscle synergies. To test this hypothesis, we investigated the linear relationships between the activities of each pair of the three heads of the triceps surae and between the plantar flexion torque and the activity of each head of the triceps surae during voluntary isometric plantar flexor contraction performed with and without voluntary isometric knee extensor contraction. Within the muscle synergy hypothesis, the relationship among the activities of the three heads of the triceps surae could be clearly described using a regression line, the slope of which can vary with knee extensor contraction. A further implication is that, when knee extensor contraction induces a change in the slope of this regression line, the change is reflected also in the slope of the regression line describing the relationship between plantar flexion torque and the activity of each head of the triceps surae. On the other hand, under the hypothesis that muscle activities can be expressed in terms of a linear combination of joint torques exerted by the muscles (rather than in terms of synergy), the slope of the regression line describing the relationship between plantar flexion torque and the activity of each head of the triceps surae is expected to remain unchanged for all levels of knee extensor contraction, because this mathematical model requires a coefficient for the plantar flexion torque (i.e., constant slope).

## Methods

### Participants

Twelve male volunteers participated in the experiment. The mean ± standard deviation values for age, height, and body mass of the participants were 24 ± 3 years, 170.8 ± 6.8 cm, and 69.4 ± 11.8 kg, respectively. No participant had a medical history of neurological disorders. All participants gave their written informed consent to participate in the study after receiving a detailed explanation of the purpose, potential benefits, and risks associated with participation. The Human Research Ethics Committee at Kanagawa University approved all procedures used in the study.

### Torque and electromyography (EMG) recordings

**Fig 1:**
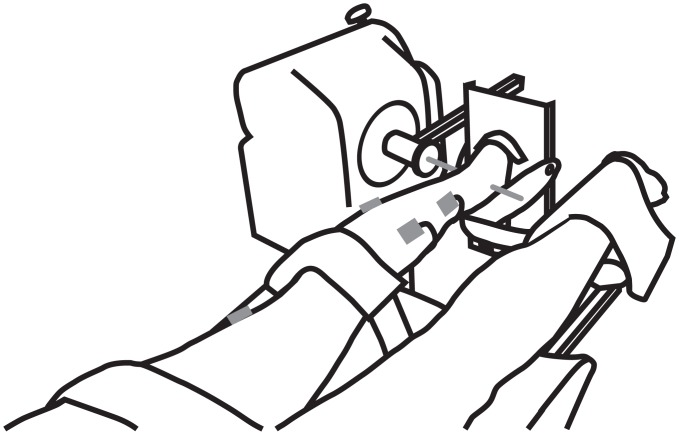
Experimental setup. The gray line represents the anatomical plantar and dorsiflexion axis. The gray rectangles represent electrodes.

The participant lay prone on a seat reclined to horizontal position, and the lower limbs were secured with straps placed around the hips and right knee to minimize fluctuations in joint angles (Fig 1). The knees were fully extended and were supported by pads that elevated the knee to prevent contact between the seat and the electrodes placed over the vastus lateralis (VL) and rectus femoris (RF) muscles. The right ankle was positioned at 0° (neutral), and the right foot was tightly fixed to the plate of a dynamometer (Biodex System 3 or Biodex System 4, Biodex Medical Systems, Shirley, NY, USA; or Cont-Rex CH-8046, CMV AG, Zürich, Switzerland). The axis of rotation of the dynamometer was aligned with the anatomical axis of ankle dorsiflexion and plantar flexion. The ankle and knee angles were kept as constant as possible throughout the experiment, to eliminate the influence of joint angles on the activity of the triceps surae [28]. These settings were maintained for all trials.

Using single differential electrodes (DE-2.1, Delsys, Boston, MA, USA), surface EMG was recorded from the VL, RF, gluteus maximus (GM), biceps femoris (BF), tibialis anterior (TA), MG, LG, and Sol of the right lower limb. After carefully abrading and cleaning the skin with alcohol, the electrodes were placed at the following sites: on the skin over the distal part of the VL, RF, and BF muscles; on the skin over the belly of the GM, TA, MG, and LG muscles; and on the skin over the medial aspect of the Sol muscle. The ground electrode was placed on the medial surface of the tibia. EMG signals were amplified (×100) using a standard biosignal recording system (Bagnoli Desktop EMG System, Delsys) and bandpass filtered at 20–450 Hz before sampling. Plantar flexion torque and EMG data were sampled at 2 kHz and stored on the hard disk of a personal computer using a 16-bit analog-to-digital converter (PowerLab 16/30 or 16/35, ADInstruments, Sydney, Australia).

### Experimental protocol

The experimental session consisted of maximal voluntary isometric contraction (MVC) trials, followed by torque-matching trials in which the participant was required to isometrically generate a constant plantar flexion torque while maintaining a constant isometric contraction of the knee extensors.

Prior to the experiment, participants practiced the task until they were able to generate the torque as intended. At the beginning of the session, participants performed MVC trials for the hip extensor, knee extensor, knee flexor, ankle plantar flexor, and ankle dorsiflexor muscles, without countermovements. Verbal encouragement was provided, and each MVC trial lasted >3 s. Two trials were performed for each muscle group. A rest period of a few minutes was provided between consecutive MVC trials. EMG was recorded during all MVC trials, but torque was measured only for the plantar flexor MVC trials. The maximum torque generated during the plantar flexor MVC trials (PFMVC) and the maximum average rectified value (ARV) of the VL EMG during the knee extensor MVC trials (KEMVC) were identified.

**Fig 2:**
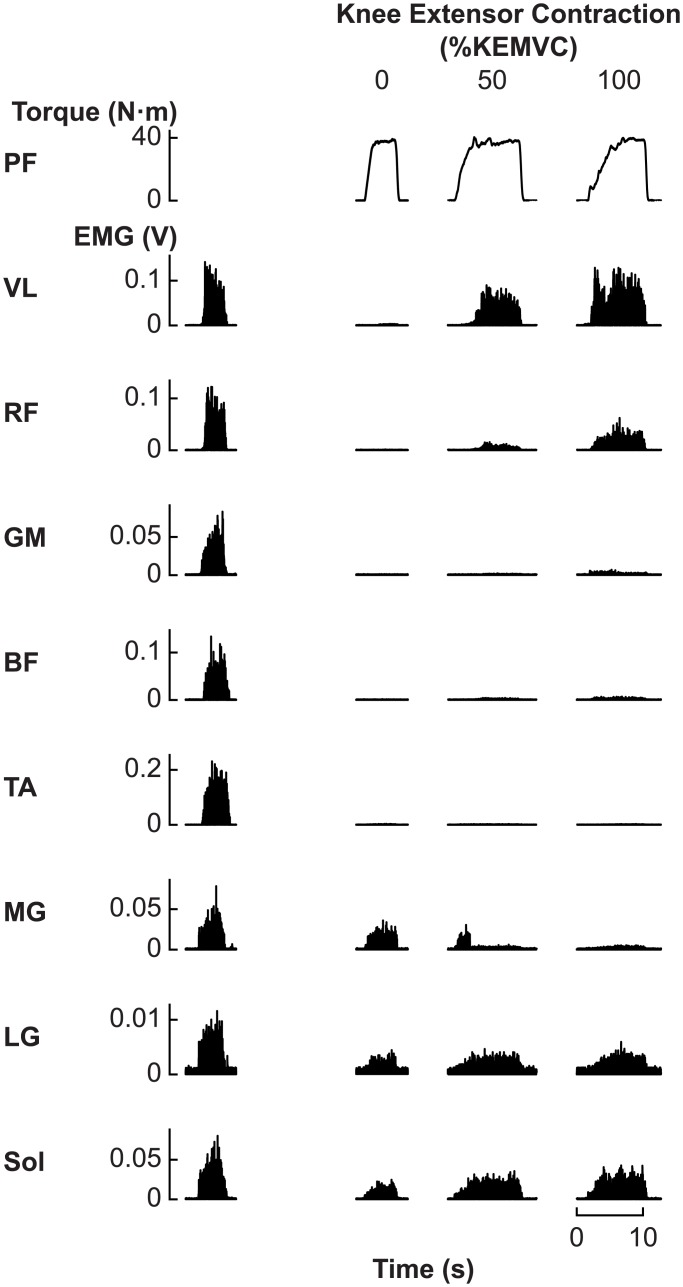
Example plantar flexion (PF) torque and electromyography (EMG) traces recorded during the experiment. *Left column*: Rectified EMG during maximal voluntary contraction for each muscle. *Other columns*: PF torque and rectified EMG during trials in which the participant had to maintain the PF torque at 40% of the maximum voluntary PF torque and the activity of the vastus lateralis (VL) muscle at 0%, 50%, or 100% of maximum (%KEMVC). EMG was recorded from the VL, rectus femoris (RF), gluteus maximus (GM), biceps femoris (BF), tibialis anterior (TA), medial gastrocnemius (MG), lateral gastrocnemius (LG), and soleus (Sol) muscles.

After a rest period of a few minutes, participants performed the torque-matching trials where they were required to isometrically generate a constant plantar flexion torque at one of ten target levels (10, 20, 30, 40, 50, 60, 70, 80, 90, or 100% PFMVC) while maintaining a constant isometric contraction of the knee extensor muscles at one of three levels (0, 50, or 100% KEMVC) (Fig 2). One trial was performed for each condition, giving a total of 30 trials (10 levels of plantar flexion torque × 3 levels of knee extensor contraction) per participant. The plantar flexion torque and knee extensor contraction conditions were presented in a random order. Each trial lasted >3 s, and the subjects were allowed a few minutes of rest between consecutive trials. The actual plantar flexion torque, target plantar flexion torque, minimally processed VL EMG signal, and target VL EMG activity level were displayed in real time on a monitor located in front of the participant. Minimal processing of the VL EMG signal involved full-wave rectification and low-pass filtering at 10 Hz, which were performed online using LabChart software (ADInstruments). Participants were also given auditory feedback regarding the level of knee extensor activity, as it would have been difficult for them to simultaneously process two forms of visual feedback. If the minimally processed VL EMG was above or below the target KEMVC by ≥10% KEMVC, the participants received auditory feedback until the processed signal returned to the target range. At 100% KEMVC, the participants were encouraged to perform a knee extensor MVC.

### Data processing

Post processing of the data was performed using in-house MATLAB algorithms (version 2014a, MathWorks, Natick, MA, USA). For the MVC trials, a 1-s analysis window was moved through the recorded data in 1/2000-s steps. For the ankle plantar flexor MVC trials, torque was averaged over each window. PFMVC torque was defined as the largest of the mean values obtained from all windows over the two trials. MVCs of the MG, LG, and Sol were taken as the ARV from the window in which PFMVC occurred. This analysis employed the widespread definition that EMG during MVC corresponds to the activity recorded at maximum torque [29]. For the MVC trials for hip extensor, knee extensor, knee flexor, and ankle dorsiflexor muscles, a 1-s analysis window was moved through the recorded data in 1/2000-s steps, and the ARV was calculated over each 1-s window. Because torque was not measured during the hip extensor, knee extensor, knee flexor, and ankle dorsiflexor MVC trials, the ARVs of the GM, VL, RF, BF, and TA during MVC were calculated as the largest of the ARVs obtained from all windows over the two MVC trials for each muscle.

For the torque-matching trials, the analysis program identified all 1-s windows where the mean error between the actual plantar flexion torque and the target plantar flexion torque was ≤1% PFMVC and then identified which of these windows showed the minimum difference between actual VL ARV and target VL ARV. In a few cases where VL ARV was substantially different from the target VL ARV, a 1-s window where VL activation was closer to the target level with +1% PFMVC error was identified. The average plantar flexion torque and the ARV of the EMG for each muscle was calculated over the chosen window and expressed as %MVC.

### Linear regression

Linear relationships between the activities of each pair of the three heads of the triceps surae, as well as the linear relationships between the exerted plantar flexion torque and the activity of each head of the triceps surae were inconsistent across the entire range of plantar flexion torques tested (see Results). Consequently, the data were divided into two sets according to the level of plantar flexion torque, with lower plantar flexion torque indicating levels of 10% to 50% PFMVC and higher plantar flexion torque indicating levels of 60% to 100% PFMVC. Linear regression analysis was applied to the muscle activities recorded for each participant. The following relationships were evaluated for each level of knee extensor contraction: the relationship between the activities of each pair of the three heads of the triceps surae (MG ARV × Sol ARV, LG ARV × Sol ARV, and MG ARV × LG ARV) at lower and higher plantar flexion torque; and the relationship between plantar flexion torque and the activity of each head of the triceps surae (PF × MG ARV, PF × LG ARV, and PF × Sol ARV) at lower and higher plantar flexion torque. For each regression line, the coefficients of regression (i.e., slope and intercept) and the coefficient of determination (*r*^2^) were calculated.

### Statistics

The ARV of VL (expressed as %MVC) at lower and higher plantar flexion torque was compared across five levels of plantar flexion torque (for lower torque, 10% to 50% PFMVC, with 10% increments; for higher torque, 60% to 100% PFMVC, with 10% increments) and three levels of knee extensor contraction (0, 50, and 100% KEMVC) using a two-way analysis of variance with repeated measures. The slope for the relationship describing the activity of each pair of heads of the triceps surae (MG × Sol, LG × Sol, and MG × LG), as well as the relationship between plantar flexion torque and the activity of each specific head of the triceps surae (PF × MG, PF × LG, and PF × Sol), was compared across knee extensor contraction levels (0, 50, and 100% KEMVC) at lower and higher plantar flexion torque using a one-way analysis of variance with repeated measures. Shaffer’s post-hoc test was conducted to examine the difference between the knee extensor contraction levels. The Greenhouse-Geisser degrees of freedom correction (ε) was used to correct for violation of the sphericity assumption. Analysis of variance and post-hoc tests were performed using statistical software (SPSS Statistics 21, IBM Japan, Tokyo, Japan). The level of significance for all comparisons was set at *P* < 0.05.

## Results

### Control of trial conditions

The mean error between the target plantar flexion torque and the actual plantar flexion torque was <1% PFMVC for all trials, with the exception of the error for trials with target 100% PFMVC/50% KEMVC, which was <1.5% PFMVC. Regardless of the target plantar flexion torque, VL ARV and RF ARV were, on average, approximately 40% MVC in trials with a 50% KEMVC target and approximately 80% MVC in trials with a 100% KEMVC target. Although VL ARV was lower than the target level, there was a significant main effect of knee extensor contraction on VL ARV at both lower plantar flexion torque (*F*_2,22_ = 614.7, ε = 0.744, *P* < 0.001) and higher plantar flexion torque (*F*_2,22_ = 874.4, ε = 0.816, *P* < 0.001). Furthermore, post hoc tests revealed a significant increase in VL ARV from 0% to 50% KEMVC, from 0% to 100% KEMVC, and from 50% to 100% KEMVC at both lower and higher plantar flexion torque (*P* < 0.001, for each comparison). GM ARV and BF ARV, on average, were <15% MVC in trials with a target of no more than 70% PFMVC; above this target plantar flexion torque, GM ARV and BF ARV were <25% MVC and <20% MVC, respectively. The mean TA ARV was <10% MVC in all trials.

### Linear relationships among the activities of the triceps surae muscles

**Fig 3:**
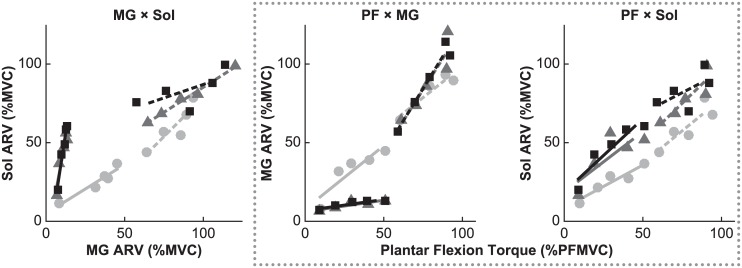
Example scatter plots for the medial gastrocnemius (MG) and soleus (Sol) electromyographic (EMG) activities. *Left*: Relationship between the average rectified value (ARV) of MG and Sol EMG activities. *Middle*: Relationship between the exerted plantar flexion torque and MG ARV. *Right*: Relationship between the exerted plantar flexion torque and Sol ARV. Data are derived from the same participant as the data given in Fig 2. Trials were performed with vastus lateralis muscle activity maintained at 0% of maximum (0% KEMVC; light gray circles), 50% of maximum (50% KEMVC; dark gray triangles), and 100% of maximum (100% KEMVC; black squares). PFMVC is the maximum torque generated during maximal voluntary contraction (MVC) of plantar flexor muscles. The ARV of the EMG is expressed as a percentage of the corresponding value during MVC (%MVC). Data points were divided according to the target plantar flexion torque and were fitted using one regression line for lower torque (10% to 50% PFMVC; solid line) and one regression line for higher torque (60% to 100% PFMVC; dashed line).

A typical scatter plot of MG and Sol ARVs across various levels of plantar flexion torque is shown in the left panel of Fig 3. At lower plantar flexion torque (i.e., 10% to 50% PFMVC), there was a clear linear relationship between the ARVs of MG and Sol at each level of knee extensor contraction; the slope of the relationship differed according to the presence of knee extensor contraction. However, at higher plantar flexion torque (i.e., 60% to 100% PFMVC), the slope was no longer dependent on the level of knee extensor contraction. The changes in the slope were first observed in a representative participant to obtain an understanding of the changes across the group; the subsequent statistical analysis of the data from all participants was performed to test whether the slope changed with the level of knee extensor contraction as observed in the representative participant.

**Fig 4:**
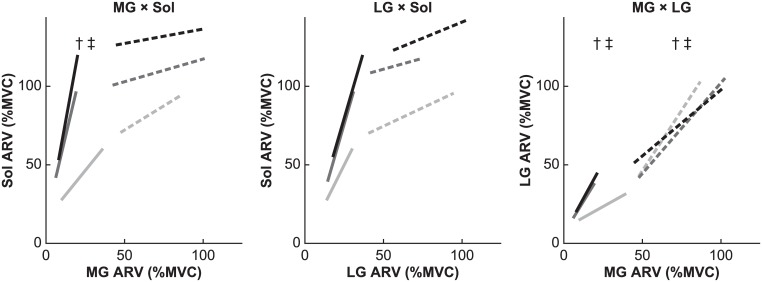
Linear relationships between electromyographic (EMG) activities during the torque-matching trials. *Left*: Mean linear regression lines for the average rectified value (ARV) of the medial gastrocnemius (MG) and soleus (Sol) EMG activities. *Middle*: Mean linear regression lines for the ARV of the lateral gastrocnemius (LG) and Sol EMG activities. *Right*: Mean linear regression lines for the ARV of MG and LG EMG activities. Trials were performed with vastus lateralis muscle activity maintained at 0% of maximum (0% KEMVC; light gray), 50% of maximum (50% KEMVC; dark gray), and 100% of maximum (100% KEMVC; black). PFMVC is the maximum torque generated during maximal voluntary contraction (MVC) of plantar flexor muscles. The ARV is expressed as a percentage of the corresponding value during MVC (%MVC). Linear regression lines, with the slope and intercept averaged for all participants, are drawn within the range of mean measured ARVs for lower plantar flexion torque (10% to 50% PFMVC; solid line) and higher plantar flexion torque (60% to 100% PFMVC; dashed line). Significant differences between regression line slopes are indicated: † *P* < 0.05 between 0% and 50% KEMVC; ‡ *P* < 0.05 between 0% and 100% KEMVC.

At lower plantar flexion torque, there was a significant main effect of knee extensor contraction on the slopes for MG × Sol (*F*_2,22_ = 8.9, ε = 0.953, *P* = 0.002) and MG × LG (*F*_2,22_ = 8.3, ε = 0.709, *P* = 0.007), but no significant main effect on the slope for LG × Sol (*F*_2,22_ = 2.5, ε = 0.530, *P* = 0.139; Fig 4 and Table 1). Shaffer’s post hoc test for MG × Sol and MG × LG revealed significant differences between 0% and 50% KEMVC (*P* = 0.009 and 0.017, respectively) and between 0% and 100% KEMVC (*P* = 0.004 and 0.008, respectively) but not between 50% and 100% KEMVC (*P* = 0.236 and 0.347, respectively). Therefore, at lower plantar flexion torque, the slopes for these pairs of triceps surae muscles differed significantly between 0% KEMVC and other levels of knee extensor activation.

**Table 1:** Linear relationships between the activities of the heads of the triceps surae at lower and higher plantar flexion torque.

Pair	%KEMVC	Lower plantar flexion torque			Higher plantar flexion torque		
		Slope	Intercept	*r*^2^	Slope	Intercept	*r*^2^
MG × Sol	0	1.2 (1.0)	15.5 (13.5)	0.66 (0.37)	0.6 (0.4)	41.4 (29.2)	0.40 (0.29)
	50	4.2 (3.3)	15.6 (34.9)	0.80 (0.21)	0.3 (0.3)	88.5 (45.3)	0.38 (0.30)
	100	5.5 (3.9)	9.1 (26.2)	0.88 (0.15)	0.2 (0.7)	118.1 (101.5)	0.42 (0.34)
LG × Sol	0	2.0 (1.1)	−0.1 (23.3)	0.76 (0.31)	0.5 (0.4)	51.1 (31.7)	0.52 (0.34)
	50	3.5 (2.6)	−10.7 (20.2)	0.91 (0.13)	0.3 (0.4)	97.0 (39.3)	0.39 (0.29)
	100	3.4 (2.4)	−6.1 (22.5)	0.89 (0.15)	0.4 (0.8)	99.9 (91.8)	0.47 (0.31)
MG × LG	0	0.6 (0.3)	9.5 (8.4)	0.74 (0.19)	1.5 (0.6)	−30.6 (48.7)	0.71 (0.22)
	50	1.6 (1.3)	6.4 (7.8)	0.83 (0.19)	1.2 (0.6)	−13.3 (58.2)	0.73 (0.22)
	100	1.8 (1.4)	5.8 (9.6)	0.87 (0.18)	0.8 (0.7)	13.6 (72.4)	0.68 (0.31)

Data are presented as mean (standard deviation) for a sample of 12 participants. Linear regression analysis was applied to the muscle activity values recorded for each participant when generating a plantar flexion torque ranging from 10% to 50% of the maximum torque (lower plantar flexion torque) and from 60% to 100% of the maximum torque (higher plantar flexion torque). MG: medial gastrocnemius; LG: lateral gastrocnemius; Sol: soleus; %KEMVC: target vastus lateralis activity as a percentage of maximum; Slope: slope of the regression line; Intercept: intercept of the regression line; *r*^2^: coefficient of determination.

At higher plantar flexion torque, *r*^2^ remained low in all trials (Table 1), indicating that the linear relationship between the two muscles in each pair was weaker. There was a significant difference in the slope for MG × LG (*F*_2,22_ = 7.0, ε = 0.699, *P* = 0.012), but no significant main effect of knee extensor contraction on the slope for MG × Sol or that for LG × Sol (*F*_2,22_ = 3.2, ε = 0.852, *P* = 0.100; *F*_2,22_ = 0.4, ε = 0.802, *P* = 0.622; respectively; Fig 4 and Table 1). Shaffer’s post hoc test for MG × LG revealed a significant decrease between 0% and 50% KEMVC (*P* = 0.034) and between 0% and 100% KEMVC (*P* = 0.015), but not between 50% and 100% KEMVC (*P* = 0.058).

### Linear relationship between plantar flexion torque and activation of each head of the triceps surae

MG ARV and Sol ARV recorded for a representative participant were replotted in the middle and right panels of Fig 3, respectively. A linear relationship between the plantar flexion torque and the ARVs of MG and Sol at each level of knee extensor contraction was detectable. According to knee extensor contraction, the slope for MG ARV was observed to decrease at lower plantar flexion torque (i.e., 10% to 50% PFMVC), but increase at higher plantar flexion torque (i.e., 60% to 100% PFMVC). The slope for Sol ARV increased with knee extensor contraction at lower plantar flexion torque. These observations noted for a representative participant were confirmed in the subsequent analysis of the same relationships in the sample including all participants.

**Fig 5:**
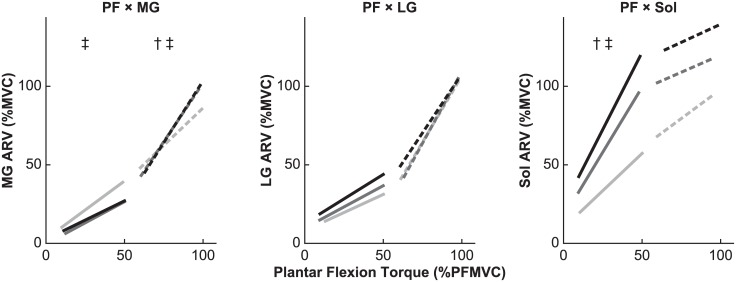
Linear relationships between plantar flexion torque and electromyographic (EMG) activity during the torque-matching trials. *Left*: Mean linear regression lines for the average rectified value (ARV) of the medial gastrocnemius (MG) EMG activity. *Middle*: Mean linear regression lines for the ARV of the lateral gastrocnemius (LG) EMG activity. *Right*: Mean linear regression lines for the ARV of the soleus (Sol) EMG activity. Trials were performed with vastus lateralis muscle activity maintained at 0% of maximum (0% KEMVC; light gray), 50% of maximum (50% KEMVC; dark gray), and 100% of maximum (100% KEMVC; black). PFMVC is the maximum torque generated during maximal voluntary contraction (MVC) of plantar flexor muscles. The ARV of the EMG is expressed as a percentage of the corresponding value during MVC (%MVC). Linear regression lines, with the slope and intercept averaged for all participants, are drawn for lower plantar flexion torque (10% to 50% PFMVC; solid line) and higher plantar flexion torque (60% to 100% PFMVC; dashed line). Significant differences between regression line slopes are indicated: † *P* < 0.05 between 0% and 50% KEMVC; ‡ *P* < 0.05 between 0% and 100% KEMVC.

At lower plantar flexion torque, there was a significant main effect of knee extensor contraction on the slope for PF × MG (*F*_2,22_ = 4.4, ε = 0.890, *P* = 0.031) and on that for PF × Sol (*F*_2,22_ = 5.2, ε = 0.521, *P* = 0.042), but no significant main effect on the slope for PF × LG (*F*_2,22_ = 1.4, ε = 0.617, *P* = 0.268; Fig 5 and Table 2). Shaffer’s post hoc test for PF × MG revealed a significant decrease between 0% and 100% KEMVC (*P* = 0.015), but not between 0% and 50% KEMVC (*P* = 0.079) or between 50% and 100% KEMVC (*P* = 0.539). Furthermore, the same post hoc analysis for PF × Sol revealed significant increase between 0% and 50% KEMVC (*P* = 0.039) and between 0% and 100% KEMVC (*P* = 0.043), but not between 50% and 100% KEMVC (*P* = 0.078).

**Table 2:** Linear relationships between plantar flexion (PF) torque and activation of each head of the triceps surae.

Muscle	%KEMVC	Lower PF torque			Higher PF torque		
		Slope	Intercept	*r*^2^	Slope	Intercept	*r*^2^
PF × MG	0	0.7 (0.3)	2.9 (6.4)	0.80 (0.17)	1.0 (0.3)	−9.1 (25.5)	0.81 (0.16)
	50	0.5 (0.4)	−0.4 (5.2)	0.86 (0.10)	1.5 (0.7)	−47.5 (58.1)	0.85 (0.10)
	100	0.5 (0.4)	2.6 (5.1)	0.87 (0.12)	1.6 (0.5)	−53.6 (47.0)	0.83 (0.11)
PF × LG	0	0.5 (0.2)	8.1 (8.4)	0.86 (0.07)	1.7 (0.5)	−62.2 (37.9)	0.84 (0.13)
	50	0.5 (0.2)	9.7 (9.2)	0.82 (0.14)	1.8 (0.7)	−72.8 (47.3)	0.83 (0.10)
	100	0.6 (0.4)	12.8 (11.0)	0.83 (0.16)	1.5 (0.8)	−41.9 (69.7)	0.78 (0.26)
PF × Sol	0	0.9 (0.4)	9.8 (12.3)	0.81 (0.24)	0.7 (0.5)	24.0 (40.5)	0.57 (0.31)
	50	1.7 (0.9)	16.7 (18.5)	0.86 (0.14)	0.4 (0.6)	76.2 (56.4)	0.40 (0.26)
	100	2.0 (1.3)	23.4 (20.8)	0.90 (0.07)	0.5 (1.0)	93.5 (127.3)	0.46 (0.33)

Data are presented as mean (standard deviation) for a sample of 12 participants. Linear regression analysis was applied to the values of muscle activity recorded for each participant when generating a PF torque ranging from 10% to 50% of the maximum torque (lower PF torque) and from 60% to 100% of the maximum torque (higher PF torque). MG: medial gastrocnemius; LG: lateral gastrocnemius; Sol: soleus; %KEMVC: target vastus lateralis activity as a percentage of maximum; Slope: slope of the regression line; Intercept: intercept of the regression line; *r*^2^: coefficient of determination.

At higher plantar flexion torque, there was a significant main effect of knee extensor contraction on the slope for PF × MG (*F*_2,22_ = 5.5, ε = 0.866, *P* = 0.016), but no significant main effect on the slope for PF × LG (*F*_2,22_ = 0.7, ε = 0.805, *P* = 0.469) or on that for PF × Sol (*F*_2,22_ = 0.7, ε = 0.778, *P* = 0.462; Fig 5 and Table 2). Shaffer’s post hoc test for PF × MG revealed a significant increase between 0% and 50% KEMVC (*P* = 0.041) and between 0% and 100% KEMVC (*P* = 0.004), but not between 50% and 100% KEMVC (*P* = 0.721).

## Discussion

### Influence of knee extensor contraction on the coactivation ratio of the triceps surae muscles

Notably, this study found that the presence of knee extensor contraction induced a change in the linear relationship between the MG and Sol activity and in the relationship between the MG and LG activity, especially during low-intensity plantar flexor contraction. These findings suggest that the existence of multiple muscle synergies, which are selectively recruited during human voluntary movements, is manifested in a task-dependent manner.

At lower plantar flexion torque, the regression line describing the activity relationship for each pair of the triceps surae heads fitted better to the data points because *r*^2^ was, on average, higher than that obtained at higher plantar flexion torque (Table 1). When knee extensor muscles were not contracting, the activity of MG, LG, and Sol, expressed as %MVC, increased with plantar flexion torque. Moreover, the activity of all three muscles increased at a similar rate (Fig 4). However, simultaneous contraction of knee extensor muscles was observed to induce a different ratio of coactivation in the triceps surae muscles during low-intensity plantar flexor contraction; interestingly, this coactivation ratio was similar for medium and maximum knee extensor activation. These results suggest that the linear relationship between the triceps surae muscles during low-intensity plantar flexor contraction is only dependent on the presence of knee extensor activation and not on the magnitude of that activation. The observed change in the slope of regression could not be described by a single muscle synergy for the plantar flexor muscles in combination with another muscle synergy for both knee extensor muscles and plantar flexor muscles. If one assumes that the latter muscle synergy increases the activity of the triceps surae muscles during additional knee extensor contraction, the intercept of the regression line is expected to change; however, the slope, which is determined by the former muscle synergy, is expected to remain unchanged. Although such a scenario might be true with respect to Sol activity at higher plantar flexion torque, the slope for MG × Sol and MG × LG was observed to change, especially at lower plantar flexion torque (Fig 4). Consequently, the fact that we obtained two clear regression lines, both observed at lower plantar flexion torque, is indicative of the presence of a minimum of two muscle synergies constraining the activity of the triceps surae muscles for plantar flexion, in combination with another synergy primarily involving the knee extensor muscles.

At higher plantar flexion torque, where *r*^2^ was lower (Table 1), the regression line for each pair of the triceps surae muscles was more ambiguous than the corresponding regression line noted at lower plantar flexion torque. One reason for this scattering is that the recruitment of only one muscle synergy results in difficulty generating substantial force; consequently, muscle synergies for the plantar flexor muscles are combined in a variable ratio. For example, Sol ARV was approximately 100% MVC on average when generating plantar flexion torque at 50% PFMVC with knee extensor contraction (Fig 4). Because the Sol ARV was the defined maximum for this condition, the recruitment of muscle synergies other than the muscle synergy that was preferred with simultaneous knee extensor contraction and the resultant activity of other plantar flexor muscles would be necessary to exceed this level of plantar flexion torque, especially with knee extensor contraction. Consequently, the recruitment of multiple muscle synergies in a variable ratio at the higher plantar flexion torque would appear as an ambiguous regression line.

Previous studies using computational decomposition techniques such as non-negative matrix factorization have also suggested a dependence of human movement on muscle synergies, but have struggled to determine the number of synergies involved [16,30]. Although many previous studies using non-negative matrix factorization set a threshold for the degree of data reconstruction, such as the variability accounted for (VAF) [31], thresholds that must be pre-specified to predict the number of muscle synergies are generally arbitrary, which can influence the results. For example, some VAF thresholds predict that, in healthy and post-stroke individuals, walking is controlled by a different number of synergies or by synergies consisting of different muscle grouping strategies; however, in the same data set, other VAF thresholds predicted the same number of muscle synergies for both healthy and post-stroke individuals, and, in some cases, even predicted similar muscle coactivation ratios [19]. Additionally, for a task requiring a participant to aim endpoint force of the arm at target levels in various directions, a recent simulation study predicted that the number of muscle synergies extracted by non-negative matrix factorization, with a VAF of either 90% (standard) or 98% (relatively high), was enough to roughly reconstruct given muscle activities, but not enough to accomplish a given performance [32]. Compared with previous studies using the computational decomposition technique, the present study provides greater clarity, as the adequate number of muscle synergies was determined using statistical significance methods. This study has corroborated the dependence of human voluntary motor control on muscle synergies.

Dimensionality reduction has been assumed as a primary implication of muscle synergies [6,7,8]. In the present study, at least two regression lines were detected for each of the two pairs of muscles (i.e., MG × Sol and MG × LG; Fig 4). Although these results did not seem to indicate dimensionality reduction, it is possible that all three heads of the triceps surae are controlled through two muscle synergies if the two regression lines for each pair of muscles could be integrated into a model describing a total of two muscle synergies, provided the ratio of LG activity to Sol activity was similar for both muscle synergies. If so, control of the triceps surae would indeed be reduced into two dimensions. Additionally, it is possible that other plantar flexor muscles such as the fibularis longus and brevis participate in the same muscle synergies; if this is the case, then dimensionality reduction is certainly present. Because the activity of other plantar flexor muscles was not recorded in this study, future research is warranted to test this possibility.

### Comparison with hypotheses unrelated to the muscle synergy hypothesis

A recent study reported that the activity of the MG, which also functions as a knee flexor, decreased with knee extensor contraction during low-intensity plantar flexor contraction; this phenomenon is seemingly in line with the concept of reciprocal inhibition, whereby antagonists are inhibited with the activity of agonists [27]. However, in this previous study, MG activity did not decrease further when knee extensor activation increased from medium to maximum level. This stagnation in MG activity beyond certain knee extensor activations was also observed in the present study (Fig 5 and Table 2). These results are, therefore, inconsistent with predictions made by the analogy of reciprocal inhibition, whereby MG activity would be expected to decrease in proportion with the activity of knee extensor muscles. In contrast, the muscle synergy hypothesis may provide a better understanding of this phenomenon. The presence of knee extensor contraction induces a change in dependent muscle synergy, which is represented by a regression line. Subsequently, MG activity decreases because the dependent muscle synergy during simultaneous knee extensor contraction requires larger contribution of the Sol than of the MG, as indicated by a slope greater than 4 for the relationship between MG and Sol activity (Fig 4 and Table 1). In a similar fashion, MG activity does not decrease with increasing knee extensor activation beyond medium levels, as dependent muscle synergy does not change. Therefore, the muscle synergy hypothesis, rather than the analogy of reciprocal inhibition, is more suitable to describe the observed change in MG activity.

In this study, the activity of the triceps surae muscles could not be approximated using a linear combination of the knee extension torque and plantar flexion torque. This is contrary to the findings of previous studies, which have reported that the activity of a given muscle at the knee and hip can be approximated using a summation of the knee extension (flexion) torque multiplied by one coefficient (i.e., a constant slope of the relation between the knee extension torque and the activity of a given muscle) and the hip extension (flexion) torque multiplied by another coefficient [25,26]. As mentioned above, the decrease in MG activity at lower plantar flexion torque plateaued with increasing knee extensor activation beyond medium levels (Fig 5 and Table 2). This result suggests that MG activity is not proportional to the knee extension torque. In addition, according to knee extensor contraction, the slope of the relationship between the plantar flexion torque and MG activity decreased at lower plantar flexion torque, but increased at higher plantar flexion torque. The slope of the relationship between the plantar flexion torque and Sol activity at lower plantar flexion torque also changed with knee extensor contraction. If MG and Sol activities had individual coefficients related to the plantar flexion torque, knee extensor contraction would not induce these changes in slope. Therefore, a linear combination of muscle synergies, rather than joint torques, has a greater explanatory capacity for the activity of the plantar flexor muscles.

The change in muscle activities observed in the present study is difficult to rationalize in the context of hypotheses based on minimization of an objective function, such as the sum of cubed muscle forces divided by the physiological cross-sectional area [23] or muscle activation [33]. In particular, the ratio of Sol activity to MG activity was approximately 1.2 during plantar flexor contraction alone, but increased to more than 4 with simultaneous knee extensor contraction (Fig 4 and Table 1). Although the sum of cubed normalized muscle forces [23] may be minimized during plantar flexor contraction alone, changes in the relationship with simultaneous knee extensor contraction would result in an excessive value of this objective function for the same plantar flexion torque, because the unbalanced muscle forces tend to increase the sum of cubes. If the increase in this slope with knee extensor contraction can minimize an objective function such as this, then the slope should also increase with increasing knee extensor activation beyond medium levels. Furthermore, it is noteworthy that the observed change in slope occurred despite maintaining the same stable posture characterized by constant length of the muscle-tendon unit, muscle contraction velocity, and moment arm, all of which influence the optimal activation of muscles [33,34]. Previous studies that have observed a single linear regression line for a pair of muscles [3,35] have been unable to deny the possibility that a single regression line was the artifact of considering the minimization hypothesis unrelated to muscle synergies [12]. This is because the minimization model has a tendency to prefer the balanced activity of muscles, leading to one regression line based on a nearly constant balance (i.e., slope). Because many previous studies performed in humans and using computational decomposition techniques did not simultaneously record joint torques or posture [13,16,19,20], it is difficult to compare the explanatory capacity of the muscle synergy hypothesis with that of the minimization hypotheses. The present study revealed that, in a constant posture, the same plantar flexion torque is generated according to at least two ratios of Sol activity to MG activity. This finding suggests that minimization hypotheses are unable to consistently predict muscle activities.

### Prospects for dynamic movements

While our finding that at least two linear regression lines may characterize certain pairs of triceps surae muscles is consistent with the muscle synergy hypothesis, these regression lines are not necessarily observed in all human movements, even if the muscle synergy hypothesis is true. The muscle synergy hypothesis allows multiple muscle synergies to be recruited in various combinations for activation of a given muscle. In these cases, clear linear regression lines between muscle activities would disappear, much like the muscle activities observed at higher plantar flexion torque in this study (Table 1). Although this study has shown evidence of muscle synergies in carefully controlled conditions, the regression analysis could not be singularly applied to complicated muscle activation, especially during dynamic movements. Nevertheless, comparison of muscle activity during dynamic movements with the results of regression analysis under static conditions has the potential to provide a valuable insight into human motor control through muscle synergies.

Information regarding the temporal organization of muscle synergies, which was lost in this study because of averaging the amplitude of EMG during the task, is important for dynamic movements. A number of previous studies have proposed a time-varying synergy, which integrates temporal components with delays [8,14,36]. A previous study evaluating arm reaching in various directions has reported that, under the assumption that a time-varying synergy contains activation patterns of multiple muscles with temporal delays, a small number of time-varying synergies could account for the activation of required muscles over a wide range of time [36]. When muscle activities were decomposed by synchronous synergy (equivalent to the muscle synergy used in this study) and time-varying synergy for comparison, the time-averaged ratio of muscle activities for a given time-varying synergy was similar to the corresponding ratio noted for synchronous synergy [14]. Therefore, it may be possible that two muscle synergies detected in the present study have temporal components, as time-varying synergies. If so, with consideration of the degrees of freedom for temporal organization, the two muscle synergies for plantar flexor muscles (as observed in the present study) would reduce dimensionality. However, it should be noted that the number of muscle synergies in this case was relatively large, which differs from many previous observations that the activities of plantar flexor muscles during walking were almost integrated into a single synergy [13,19,20]. Temporal organization of muscle synergies detected in the present study, and the practical significance of these synergies during dynamic movements, will require further research.

## Conclusions

This study has revealed that the presence of knee extensor contraction induces a change in the linear relationship among the activities of the three heads of the triceps surae, especially with constant low plantar flexion torque, resulting in a change in the linear relationship between the plantar flexion torque and the activity of the MG or the Sol. These findings, which are consistent with the muscle synergy hypothesis, provide behavioral evidence that muscle synergies, characterized by non-mechanical constraints, are selectively recruited during human voluntary movements in a manner relevant to the task to be accomplished.

## Supporting information

S1 Table Participant-specific linear relationships among the activities of the heads of the triceps surae and between plantar flexion (PF) torque and activity of each head of the triceps surae. Linear regression analysis was applied to the muscle activities recorded for each of the 12 participants when generating a PF torque ranging from 10% to 50% of the maximum torque (lower PF torque) and from 60% to 100% of the maximum torque (higher PF torque). MG: medial gastrocnemius; LG: lateral gastrocnemius; Sol: soleus; %KEMVC: target vastus lateralis activity as a percentage of the maximum; Slope: slope of the regression line; Intercept: intercept of the regression line; *r*^2^: coefficient of determination.
(XLSX)
